# HTLV-2 in Central Africa: HTLV-2 subtype B strains similar to those found in Amerindian tribes are endemic in Bakola Pygmies from south Cameroon but not in surrounding Bantus and Baka Pygmies

**DOI:** 10.1186/1742-4690-8-S1-A82

**Published:** 2011-06-06

**Authors:** Philippe Mauclère, Laurent Meertens, Philippe Afonso, Sabine Plancoulaine, Claudia Filippone, Edouard Betsem, Sara Calattini, Alain Froment, Monique Van Beveren, Guy de Thé, Luis Quintana-Murci, Renaud Mahieux, Antoine Gessain

**Affiliations:** 1 Unité EPVO, CNRS URA 3015, Institut Pasteur, Paris, France; 2 Centre Pasteur du Cameroun, Yaoundé, Cameroun; 3 Laboratoire GHMI, INSERM, U.550, Paris, France; 4 Faculté de Médecine et des Sciences Biomédicales, Université de Yaoundé, Cameroun; 5 IRD, Musée de l’Homme, Paris, France; 6 HEG Unit, CNRS URA 3012, Institut Pasteur, Paris, France

## Background

Presence and origin of endemic foci of HTLV-2 infection in Africa remain a matter of debate.

## Material and methods

To better appreciate the epidemiological and molecular determinants of HTLV-2 infection in Central Africa, we performed a survey in 3903 inhabitants of a South Cameroon forest area, including 1051 Bakola Pygmies, 815 Baka Pygmies and 2037 Bantus living in their neighboring. HTLV-1 and HTLV-2 infection was determined by both specific serological (IFA and WB) and molecular (different generic and specific PCR) methods.

## Results

HTLV-1/2 prevalence was of 3% (117/3903) with 90 HTLV-1 (2.3%) and 27 HTLV-2 (0.7%). Surprisingly, HTLV-2 infection was restricted to Bakola Pygmies (27/1051 2.5%) with no HTLV-2 infection in any of the 2852 Baka or Bantus individuals. In Bakola Pygmies, HTLV-2 seroprevalence increased with age, reaching 6.5% in the elder persons. Ongoing intrafamilial HTLV-2 transmission was evidenced. Lymphoid T cell lines (CD8+ or CD4+, CD25 +) producing HTLV-2 antigens, were established from PBMCs cultures of HTLV-2 infected individuals. Sequences of a 672 nucleotide LTR fragment, obtained from 7 HTLV-2 samples, showed a very high degree of homologies among samples (< 1% nucleotide divergence) but also surprisingly with Amerindian HTLV-2 B strains. Complete sequence (8954 bp) of one isolate confirmed a typical HTLV-2 B strain.

## Conclusion

This study demonstrates clearly a HTLV-2 endemic population, with ongoing transmission, in Central Africa. Furthermore, it gives insights into several central questions regarding the origin and evolution rate of HTLV-2 and the migrations of infected populations.

